# Cord-lamina angle and foraminal diameter as key predictors of C5 palsy after anterior cervical decompression and fusion surgery

**DOI:** 10.1515/med-2025-1162

**Published:** 2025-05-07

**Authors:** Wenchao Zhou, Baifeng Sun, Zichuan Wu, Aochen Xu, Hanlin Song, Xuhong Zhang, Junbin Liu, Junzhe Sheng, Yang Liu

**Affiliations:** Department of Spinal Surgery, No. 905 Hospital of PLA Navy, Shanghai, China; Department of Spinal Surgery, Changzheng Hospital Affiliated to Navy Medical University, Shanghai, China

**Keywords:** C5 palsy, risk factor, CLA, anterior cervical surgery, cervical spine

## Abstract

**Background:**

C5 nerve root palsy (C5P) is a rare but documented complication after cervical decompression surgery. Despite multiple proposed mechanisms, research on risk factors for C5P after anterior cervical discectomy and fusion (ACDF) remains insufficient.

**Objective:**

The aim of this study was to identify specific risk factors associated with C5 nerve root palsy after ACDF.

**Methods:**

A total of 108 patients who underwent ACDF surgery were divided into C5P and non-C5P groups. The basic data, preoperative Japanese Orthopaedic Association score, cervical curvature correction at C2–7 and C4–5, anterior–posterior diameter (APD) at C4/5, spinal-cord-lumbar angle (CLA) at C4/5, foramen magnum occipitalis at C4/5, and the presence of preoperative high-signal areas in the C4/5 spinal cord on T2-weighted magnetic resonance imaging, were present. Risk factors associated with C5P were identified using logistic regression analysis.

**Results:**

There were no significant differences between the two groups in terms of age, gender, disease duration, diagnosis, preoperative Japanese Orthopaedic Association score, number of operated segments, APD, or high-intensity zone. LRA showed that a larger CLA and narrower FD at C4/5 were the main risk factors for the development of C5P (*P* < 0.05).

**Conclusion:**

A larger cord-lamina angle and a narrower foraminal diameter at C4/5 are significant risk factors associated with the development of C5 palsy following ACDF.

## 1 Introduction

Cervical spondylosis is one of the most common cervical degenerative diseases. In cervical spondylosis, disc herniation, ligamentum luteum, posterior longitudinal ossification, and other compression of cervical pulp and nerves are the main reasons for patients’ decreased working ability and living standards [1]. The incidence of cervical spondylosis in people under the age of 25 is 20–25%, and the incidence of cervical spondylosis in people over the age of 65 is as high as 65–70%. The onset of cervical spondylosis is slow and acute attacks rarely occur [2]. Therefore, for mild non-progressive cervical spondylosis patients, conservative treatment can generally be taken, but for acute patients with severe neurological dysfunction, cervical decompression surgery is often the final treatment. Cervical decompression procedures include anterior cervical decompression fusion (ADF) and posterior cervical decompression fusion (PDF): anterior cervical discectomy and fusion (ACDF), anterior cervical subtotal vertebral fusion, combined anterior and posterior cervical surgery (hybrid), laminoplasty (LP), and posterior laminectomy and fusion [3,4]. In both ADF and PDF procedures, complications of C5 root paralysis (C5P) are possible. Therefore, it is urgent to clarify the key predictors of C5P.

C5 palsy (C5P) is a rare yet recognized complication of cervical spine decompression surgery, characterized by weakness or paralysis of the deltoid and/or biceps brachii muscles [5]. This condition is typically identified using the manual muscle test (MMT) score, where C5P presents as a decrease of at least one grade compared to the preoperative score [6,7]. Although the incidence of C5P is low [5,8], it can lead to a diminished quality of life and significant healthcare costs. A study conducted a study comparing the anterior and posterior surgical approaches and found that the occurrence of C5 palsy was considerably higher when using the posterior approach (8.6%) compared to the anterior approach (1.6%), with a statistically significant difference (*P* < 0.001) [9]. A separate study indicated that the incidence of C5 palsy was reported to be 0.7% for the anterior approach and 8.8% for the posterior approach [10]. Additionally, another study reported that the incidence of C5 palsy after posterior surgery was 24.3%, whereas there were no cases of C5 palsy following anterior surgery [11]. These findings highlight the importance of considering a surgical approach when assessing the risk of C5 palsy. The etiology of C5P is complex and may arise from both anterior and posterior surgical approaches. While various hypotheses have been proposed to explain the mechanism of postoperative C5 palsy, research specifically examining anterior C5 palsy remains limited [12]. The anatomical factors, incidence, and mechanisms underlying C5P are not yet fully understood. Therefore, this study aims to identify risk factors associated with the development of C5 palsy following ACDF.

## 2 Materials and methods

### 2.1 Patient population

Overall 108 successive patients who underwent ACDF consisting of the C4–C5 disc level from April 5, 2019, to July 5, 2021, at our hospital were retrospectively studied. Exclusion measures were as follows: (1) in the MMT, at grade 3 or less preoperative deltoid muscle deterioration, (2) trauma, (3) tumor, (4) infection, (5) fracture in cervical vertebrae, (6) cervical surgery history, (7) lack of adequate follow-up data, and (8) severe systemic disease or osteoporosis. Eventually, 108 patients (45 males and 63 females) were added to the research.

### 2.2 Surgical procedures

A single experienced surgeon performed ACDF on all patients. Following general anaesthetic induction using a right anterior approach, ACDF was executed. The surgeon used a Kerrison rongeur to excise all tissues within the intervertebral disc, and a curette or burr was utilized to abrade the endplates to ensure adequate neural decompression. Subsequently, a polyether ketone cage filled with cancellous bone was inserted into the disc space, followed by the application of an anterior plate system. Postoperatively, patients were instructed to wear cervical collars for a duration ranging from 2 to 8 weeks. The postoperative cervical collar-wearing period ranged from 2 to 8 weeks, as determined by the treating surgeon based on individual patient factors such as the specific surgical procedure, healing process, severity of preoperative symptoms, and surgeon’s postoperative protocol. This variability reflects the individualized approach to care in our study.

### 2.3 Clinical and radiological assessment

The age, sex, course and diagnosis of disease, number of surgical levels, and preoperative Japanese Orthopaedic Association (JOA) scores among the two groups were evaluated.

### 2.4 Measurement of cervical lordosis angle (CLA)

The CLA was measured using standard radiographic techniques. Specifically, lateral cervical radiographs were obtained for all participants with the head in a neutral position. The CLA was then determined by drawing a line along the inferior endplate of C2 and another line along the inferior endplate of C7. The angle between these two lines was measured using digital imaging software. To ensure accuracy, all measurements were performed by two independent observers. Inter-observer reliability was assessed using the intraclass correlation coefficient (ICC), and the results demonstrated high agreement between observers (ICC > 0.85). Additionally, intra-observer reliability was assessed by having each observer repeat the measurements on a subset of radiographs at different time points. The ICC values for intra-observer reliability were also high, indicating consistent and reliable measurements.

### 2.5 Measurement of facet joint distance (FD)

FD was measured on axial cervical radiographs. The distance between the inferior articular processes of adjacent cervical vertebrae was identified and measured using digital imaging software. To achieve this, clear landmarks were identified on each radiograph, and measurements were taken perpendicular to the line connecting these landmarks. Similar to the CLA measurements, two independent observers performed all FD measurements. Inter-observer reliability was again assessed using the ICC, and the results showed excellent agreement between observers (ICC > 0.90). Intra-observer reliability was also evaluated by having each observer repeat the measurements on a different subset of radiographs. The high ICC values obtained for intra-observer reliability further confirm the consistency and reliability of the FD measurements.

### 2.6 Statistical analysis

Statistical analyses were performed by using the SPSS 22.0 statistical software program (SPSS, Inc., Chicago, IL, USA). The variations among C5P group and non-C5P group measurements were analyzed. Using an independent *t*-test or chi-squared test, recognize remarkable variations among two groups. Because the values were presumed to be relevant biological measurements; hence, the minimum FD and the maximum CLA value were employed for the logistic regression [31]. Multivariate LRA was employed for the analysis of associative risk factors. Using univariate analysis, the parameter significance. Factors with *P* < 0.20 in univariate analysis were further added to the multivariate analysis. All two-sided statistical tests and *P* < 0.05 were ascertained as statistically significant. Significance was regarded as *P* < 0.05 in all analyses. Results were exhibited as mean ± standard deviation.


**Informed consent:** All patients obtained written informed consent.
**Ethical approval:** This study protocol was approved by the Ethics Committee of Changzheng Hospital Affiliated with Naval Medical University (No. czsg22).

## 3 Results

Of the 108 patients, 45 were male and 63 were female. Patients ranged in age from 33 to 73 years with a mean age of 55.03 years and a standard deviation of 8.61 years. The duration of the disease ranged from 1 to 16 months, with a mean duration of 9.26 months and a standard deviation of 3.23 months. In terms of diagnosis, there were 43 cases of cervical spinal stenosis (CSM), 22 cases of cervical spinal stenosis with degenerative disease (CSMR), 26 cases of cervical posterior longitudinal ligament ossification (CSR), and 17 cases of other diagnoses. The comparison of patient characteristics between the C5P group and the non-C5P group is presented in Table 1. A total of 8 patients were identified with C5P, and 100 patients were in the Non-C5P group. There were no significant differences in age (56.50 ± 5.86 vs 54.91 ± 8.81 years, *P* = 0.75), duration of disease (10.50 ± 3.50 vs 9.16 ± 3.21 months, *P* = 0.26), number of surgical levels (2.38 ± 0.92 vs 2.58 ± 0.64, *P* = 0.40), or preoperative JOA score (11.75 ± 1.28 vs 12.22 ± 1.26, *P* = 0.31) between the two groups. In terms of sex distribution, 3 males and 5 females were in the C5P group, compared to 42 males and 58 females in the non-C5P group (*P* = 0.80). Regarding diagnosis, 3 patients in the C5P group and 40 patients in the Non-C5P group had CSM (*P* = 0.79), 2 vs 20 had CSMR, 1 vs 25 had CSR, and 2 vs 15 had OPLL. The results suggest that there are no significant differences in patient characteristics, including age, sex, diagnosis, duration of disease, number of surgical levels, and preoperative JOA score, between patients with and without postoperative C5P.

**Table 1:** Statistical summary in 108 patients

Item	Patients	C5P	Non-C5P	*P* value
Sex				
Male	45	3	42	0.80
Female	63	5	58	
Total	108	8	100	
Age (years)	33–73 (55.03 ± 8.61)	56.50 ± 5.86	54.91 ± 8.81	0.75
Duration of disease (months)	1–16 (9.26 ± 3.23)	10.50 ± 3.50	9.16 ± 3.21	0.26
Diagnosis				
CSM	43	3	40	0.79
CSMR	22	2	20	
CSR	26	1	25	
OPLL	17	2	15	
Surgery levels				
Level 1	7			
Level 2	36			
Level 3	62			
Level 4	3			
No. surgical levels		2.38 ± 0.92	2.58 ± 0.64	0.40
Preoperative JOA score (points)	12.18 ± 1.26	11.75 ± 1.28	12.22 ± 1.26	0.31
Follow-up period (m)	3–36 (13.62 ± 7.11)			

C5P, C5 palsy; non-C5P, non-C5 palsy; CSM, cervical spondylotic myelopathy; CSMR, cervical spondylotic myeloradiculopathy; CSR, cervical spondylotic radiculopathy; OPLL, ossification of posterior longitudinal ligament; JOA, Japanese Orthopaedic Association.

Table 2 presents detailed information on the eight patients who developed postoperative C5P following anterior cervical decompression and fusion, including their demographic characteristics (age, sex), clinical diagnosis, number of fusion segments, time to onset of paralysis symptoms after surgery (in days), and both preoperative and final MTT results. This data allows us to analyze the potential relationships between these factors and the occurrence of C5P.

Table 2 shows that the eight patients with C5P are middle-aged and elderly, and the female patients are slightly more than the male patients. In terms of diagnosis, CSM, OPLL (posterior longitudinal ligament ossification), CSR (cervical posterior longitudinal ligament ossification with cervical spondylotic myelopathy), and CSMR (multilevel cervical spondylotic myelopathy with posterior longitudinal ligament ossification) are all involved. In terms of the number of fusion segments, the number of patients with three-segment fusion was the largest, accounting for 50%. The time between the onset of paralysis symptoms varies from 0 to 3 days after surgery, indicating that C5P symptoms may appear immediately after surgery or within a few days after surgery. The majority of patients with both preoperative and final MTT results was 5, which may be related to the scoring standard of MTT and the specific conditions of patients.

**Table 2:** 8 patients with postoperative C5P characteristics

Case	Age	Sex	Diagnosis	Levels of fusion	Time from surgery to paralysis (days)	Pre-MTT	Final-MTT
1	56	F	CSM	1	0	5	5
2	59	F	OPLL	3	2	5	5
3	48	F	CSM	3	1	5	5
4	58	F	CSR	3	1	5	5
5	60	M	CSMR	3	2	5	4
6	56	M	CSM	3	3	5	5
7	49	F	OPLL	2	0	5	4
8	66	M	CSMR	1	1	5	5

CSM: cervical spondylotic myelopathy; OPLL: ossification of the posterior longitudinal ligament; CSR: cervical spondylotic radiculopathy; CSMR: cervical spondylotic myelopathy with radiculopathy.

To compare the radiological measurements between patients with and without postoperative C5 palsy (C5P). Table 3 presents the comparison of radiological measurements between the C5P and non-C5P groups. Significant differences were observed in the C2–7 Cobb correction (*P* = 0.03) and CLA (*P* < 0.001), with the C5P group demonstrating greater correction and lordosis, respectively. Additionally, there were significant differences in FD (*P* < 0.001), with the C5P group having a smaller distance compared to the non-C5P group. However, no significant differences were found in C4–5 Cobb correction (*P* = 0.24), anterior-posterior diameter (APD) (*P* = 0.31), or the presence of preoperative high-intensity zone (HIZ) in T2-weighted magnetic resonance imaging (T2W MRI) at C4/5 (*P* = 0.50) between the two groups.

**Table 3:** Comparison of the radiological measurements presence and absence of postoperative C5P

Measurements	C5P	Non-C5P	*P* value
C2–7 Cobb correction (°)	11.12 ± 4.06	5.45 ± 7.04	0.03*
C4–5 Cobb correction (°)	2.96 ± 1.32	1.45 ± 3.61	0.24
Preoperative HIZ in T2-weighted MRI at C4/5			
Have	5	50	0.50
Not have	3	50	
APD (mm)	7.26 ± 2.14	7.81 ± 1.39	0.31
CLA (°)	46.90 ± 5.45	38.63 ± 5.84	<0.001*
FD (mm)	2.60 ± 0.72	3.34 ± 0.61	<0.001*

C5P: C5 palsy, C5; Cobb: Cobb angle; HIZ: high-intensity zone; MRI: magnetic resonance imaging; APD: anterior–posterior diameter; CLA: cervical lordosis angle; FD: foraminal diameter.

Lastly, the logistic regression analysis for the risk factors of postoperative C5P revealed significant associations (Table 4). Specifically, in the univariate analysis, only the CLA variable showed a statistically significant relationship with postoperative C5P, with a risk ratio (RR) of 1.39 (95% confidence interval [CI]: 1.04–1.85, *P* = 0.03). In the multivariate analysis, both CLA and FD were found to be significant predictors of postoperative C5P. The RR for CLA was 1.36 (95% CI: 1.04–1.77, *P* = 0.02), indicating an increased risk of postoperative C5P. Similarly, the RR for FD was 0.11 (95% CI: 0.01–0.84, *P* = 0.03), suggesting a protective effect against postoperative C5P. However, the other variables, including age, sex, diagnosis, course of disease, number of surgical levels, preoperative JOA score, C2–7 Cobb correction, C4–5 Cobb correction, APD, and preoperative HIZ in T2W MRI at C4/5, did not show statistically significant associations with postoperative C5P in either the univariate or multivariate analyses. These findings underscore the clinical significance of our logistic regression analysis for postoperative C5 palsy (C5P). The identification of CLA as a significant risk factor in both univariate and multivariate analyses highlights its potential role in predicting patients at higher risk for developing this complication. The risk ratio of 1.36 for CLA, with a 95% CI of 1.04–1.77 and a *P*-value of 0.02, emphasizes that patients with increased CLA values may face a notably heightened likelihood of experiencing postoperative C5P.

**Table 4:** Logistic regression analysis for the risk factors of postoperative C5P

Variables	Univariate		Multivariate		Cutoff value
	RR (95% CI)	*P* value	RR (95% CI)	*P* value	
Age	1.00 (0.84–1.20)	0.34			
Sex	1.23 (0.08–19.26)	0.88			
Diagnosis	0.81 (0.26–2.52)	0.71	0.88 (0.32–2.44)	0.80	
Course of disease	1.10 (0.74–1.64)	0.64			
No. surgical levels	0.40 (0.05–3.45)	0.40			
Preoperative JOA score	0.68 (0.20–2.31)	0.54			
C2–7 Cobb correction	1.14 (0.91–1.44)	0.27	1.14 (0.95–1.36)	0.18	
C4–5 Cobb correction	1.04 (0.68–1.58)	0.86			
APD	0.42 (0.15–1.14)	0.09	0.48 (0.23–1.03)	0.06	
CLA	1.39 (1.04–1.85)	0.03	1.36 (1.04–1.77)	0.02	1.37
FD	0.10 (0.01–1.16)	0.07	0.11 (0.01–0.84)	0.03	0.42
Preoperative HIZ in T2W MRI at C4/5	0.64 (0.04–10.31)	0.75			

Conversely, the protective effect of FD, with a risk ratio of 0.11 and a 95% CI of 0.01–0.84 (*P* = 0.03), suggests that interventions aimed at enhancing FD could potentially mitigate the risk of postoperative C5P. This protective association underscores the importance of considering FD in preoperative planning and risk stratification for spinal surgeries.

The lack of statistically significant associations between other variables, such as age, sex, diagnosis, preoperative clinical indicators, and postoperative C5P further emphasizes the specificity of our findings. The robustness of our analysis, which controlled for multiple potential confounders, underscores the clinical relevance of focusing on CLA and FD in assessing and potentially mitigating the risk of postoperative C5P. These insights can inform preoperative risk assessments, surgical decision-making, and postoperative management strategies, ultimately aiming to improve patient outcomes following spinal surgeries.

## 4 Discussion

ACDF is a widely employed surgical technique for treating various cervical degenerative conditions due to its effectiveness in correcting cervical deformity and achieving direct decompression [13,14]. However, despite its benefits, ACDF is associated with certain postoperative complications, notably C5 palsy (C5P). This complication can arise from both anterior and posterior surgical approaches. Previous studies have reported a prevalence of C5P in ACDF patients ranging from 0 to 12% [15–17]. While our findings suggest that patients undergoing anterior approaches may have a lower risk of developing C5P, other studies have shown conflicting results. For instance, there was no significant difference in C5P incidence between anterior and posterior approaches [18]. Similarly, a review of cervical surgery complications from January 1970 to February 2015 reported C5P rates of 6.35% ± 5.39% for posterior approaches and 4.98% ± 3.80% for anterior approaches, indicating no significant difference between the two surgical routes [19].

Several theories regarding the causes of postoperative C5P following ACDF. One is direct damage in the C5 root because of the surgical procedure or drilling by frictional heat [20]. The theory holds the opinion that intraoperative iatrogenic damage to the C5 nerve root is the important reason for C5P. Recently, Pennington [21] has reviewed 100 consecutive patients undergoing laminectomy/LP. Each patient’s intraoperative somatosensory evoked potentials, electromyography, and transcranial motor evoked potentials were studied and examined. Results showed that a possible warning sign of nerve root irritation or injury is the sustained EMG activity of the C5 nerve root during surgery. If this mechanism is established, why delayed anterior C5 palsy happen? Imagama recommended that the postoperative C5P development is uncorrelated to the spinal cord intraoperative injury or nerve root because, in transcranial electric motor-evoked potential monitoring during surgery, there were no abnormal events were found [5]. So this theory remains open to question.

This hypothesis suggests that traction-induced lesions in the nerve root may result from a posterior shift of the spinal cord, particularly at the C5 segment, where the cord displacement is typically most pronounced in LP procedures. The nerve root anchoring at the uncovertebral joint and/or the superior facet edge may contribute to this unraveling phenomenon [22]. Although this explanation accounts for cases in posterior decompression surgeries, it does not clarify why C5 palsy also occurs following anterior approaches [23].

Another theory posits that damage to the anterior horn cells in the gray matter of the spinal cord may contribute to paralysis in the postoperative segmental motor area. This hypothesis is supported by the observation of highly intense areas in the spinal cord on postoperative T2W MRI in patients with C5 palsy [24]. However, a study found no significant differences in the occurrence of these areas between patients with and without C5 palsy, noting that highly intense areas are often located in the central region of the cervical spine [25].

From the above discussion, it is evident that there is no definitive etiology responsible for C5 palsy, particularly for anterior C5 palsy. Therefore, identifying risk factors that contribute to this serious complication is essential to prevent it as much as possible. A study conducted a retrospective study comparing global lordosis and local lordosis pre- and post-ACDF [26]. Their findings revealed that enhanced cervical spinal column lordosis can lead to spinal cord and C5 nerve root traction injuries. Our results indicate that the correction of C2–7 curvature is more pronounced in the C5 palsy group compared to the non-C5 palsy group, which aligns with previous studies. C5 palsy may enhance cervical lordosis due to the compressive forces required for lordotic alignment, which can also result in iatrogenic foraminal stenosis [27,28]. The research outcomes demonstrated significant differences in C2–7 curvature correction but not in C4–5 curvature correction. Multivariate logistic regression analysis did not show significance with either correction, possibly due to the insufficient sample size of the C5 palsy group. Further collection of C5 palsy cases is necessary to minimize the mean error.

The logistic regression analysis conducted in Table 4 provides valuable insights into the potential risk factors for postoperative C5P. Besides the significant factors identified, such as CLA and FD, it is crucial to explore the association between other radiographic parameters and C5P, especially considering their clinical relevance. First, age, sex, course of disease, number of surgical levels, and preoperative JOA score were not found to be significantly associated with C5P in either univariate or multivariate analyses. These results suggest that these parameters may not play a direct role in the development of C5P following surgery. However, it is important to note that the lack of significance could also be due to the sample size or other confounding factors not accounted for in this study. Regarding the cervical lordosis corrections, specifically C2–7 Cobb correction and C4–5 Cobb correction, their associations with C5P were not statistically significant in the multivariate analysis, despite a slight trend towards significance for C2–7 Cobb correction in the univariate analysis. This finding implies that correcting cervical lordosis may not necessarily reduce the risk of C5P. However, it is essential to consider individual patient variability and surgical indications when interpreting these results, as cervical lordosis correction is often crucial for achieving optimal surgical outcomes. Additionally, the association between APD and C5P approached statistical significance in both univariate and multivariate analyses, with *P*-values of 0.09 and 0.06, respectively. Although these results did not reach the conventional level of significance, they suggest a potential trend that warrants further investigation. The close proximity of APD to significance highlights the need for larger studies to confirm or refute its role as a risk factor for C5P.

**Figure 1:**
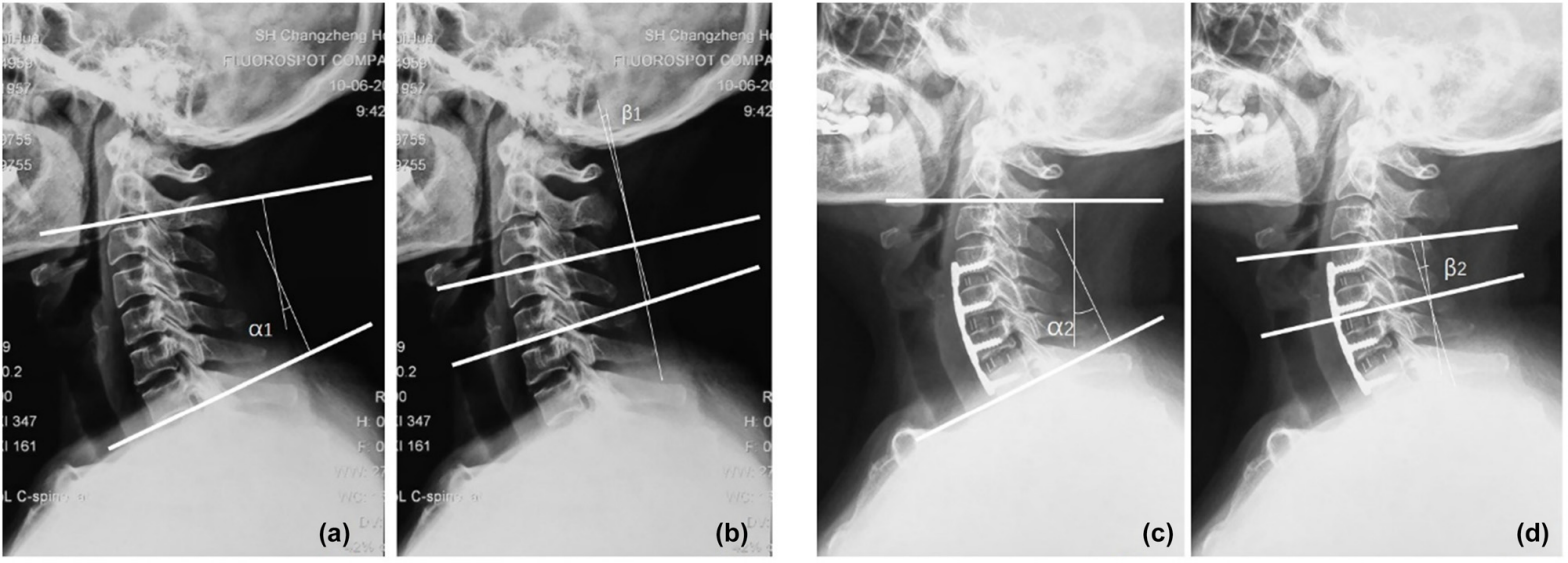
Preoperative C2–7 cervical curvature = α1 (a), preoperative C4–5 cervical curvature = β1 (b), postoperative C2–7 cervical curvature = α2 (c), and postoperative C4–5 cervical curvature = β1 (d).

**Figure 2:**
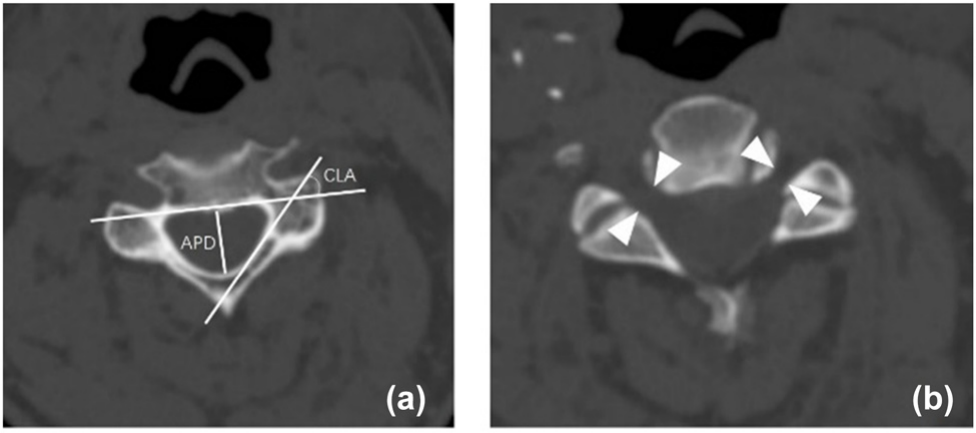
Preoperative APD, cord-lamina angle of C4/5 (CLA) (a). Foraminal diameter of C4/5 (FD) (b).

Several studies have highlighted the strong predictive value of C4/5 foraminal stenosis in the occurrence of C5 palsy, which may be linked to C5 nerve root ischemic/reperfusion injury following LP [29]. In our cohort of 102 patients who underwent LP, 16 developed C5 palsy, and the risk of C5 palsy increased substantially in patients with an intervertebral foramen thickness of less than 2.57 mm. Similarly, C4/5 foraminal stenosis has been significantly associated with C5 palsy following ACDF in several retrospective studies [30,31]. These findings suggest that while prophylactic foraminotomy may reduce the incidence of C5 palsy, it does not eliminate the risk entirely (Figures 1 and 2).

**Figure 3:**
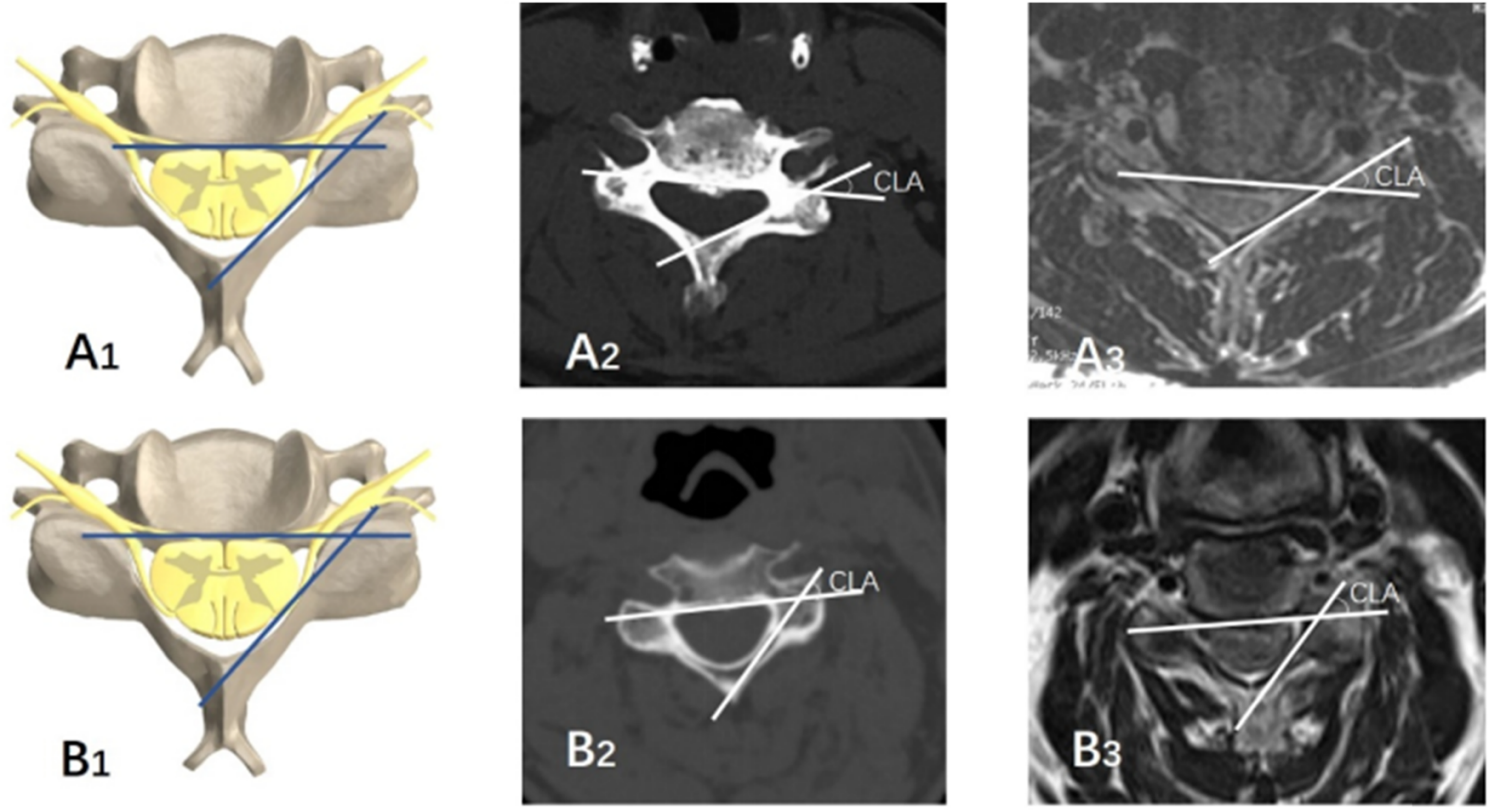
Smaller cord-lamina angle (A1, A2, A3) of C5 palsy patient and larger cord-lamina angle (B1, B2, B3) of non-C5 palsy patient.

Our findings are in line with previous reports, which indicate that ACDF can expand the foramen through the intervertebral space, potentially injuring the nerve roots with the Kerrison rongeur. The occurrence of foraminal stenosis leading to C5 palsy can be explained by two mechanisms: direct C5 root injury, such as thermal injury during foraminotomy, and ischemic changes in the decompressed C5 roots that are already predisposed to palsy. It is worth noting that spinal cord impairment might also be a contributing factor to paralysis, as some patients developed C5 palsy despite undergoing prophylactic C4/5 foraminotomy and often had preoperative compression at the C4/5 level of the spinal cord [22]. A study found that for every 1-mm increase in APD and FD, the likelihood of palsy development decreased by 69% (*P* < 0.0001) and 98% (*P* < 0.0003), respectively [32]. They suggested that greater CLA and APD following decompression could result in posterior bowing and stretching of the nerve roots. Patients with higher CLA are theoretically more susceptible to C5 root tethering following posterior and possibly anterior decompressions. Our research indicates that the average CLA in the C5 palsy group was 46.90°, while in the non-C5 palsy group, it was 38.63° (Figure 3). This difference is statistically significant. We hypothesize that a narrower FD causes chronic extrusion of the nerve root, making it more vulnerable to damage. During anterior surgery, the Kerrison rongeur is often used to remove additional spurs in the nerve root canal through the intervertebral space, which can inevitably injure the cervical spine and nerve roots. CLA indirectly reflects the pathway of the nerve roots; a larger CLA indicates a larger angle between the nerve root and the posterior border of the vertebra. When the rongeur is inserted, nerve roots with a larger CLA experience greater deformation, making them more susceptible to injury and potentially explaining the occurrence of C5 palsy.

Our study faced several limitations. First, the sample size of the C5 palsy group was insufficient to generate definitive results. Second, as a retrospective study, we could only establish correlations among anatomical factors and not causation. To confirm that smaller FD and larger CLA are risk factors for C5 palsy, a larger patient cohort and controlled experiments are needed. Additionally, basic experimental studies are required to demonstrate that ischemia–reperfusion injury and nerve fiber pathways are responsible for C5 palsy. A final key limitation of this study is the relatively small sample size, particularly the low number of C5P cases. This limited statistical power may have obscured important associations between potential risk factors and the occurrence of C5P. As a result, some factors that could be truly relevant may not have shown significance due to the insufficient number of events to detect a difference. Future studies with larger sample sizes are needed to confirm the findings and explore additional potential risk factors more comprehensively.

In the future study, we plan to carry out the following work: first, to conduct a multi-center study to verify our research results more widely; second, to track the long-term prognosis of C5P patients and analyze its development trend in depth; and third, to explore effective prevention strategies based on CLA and FD optimization. These studies will further enrich our understanding of C5P and related areas to provide more effective treatment options for patients.

## 5 Conclusion

Greater cord-lamina angle and a narrower C4/5 foramen diameter in ACDF patients are associated risk factors for C5 palsy, which can be linked to ischemic/reperfusion injury. Surgeons should consider adopting gentle surgical techniques to minimize the occurrence of C5 palsy.
